# K-Carrageenan Stimulates Pre-Osteoblast Proliferation and Osteogenic Differentiation: A Potential Factor for the Promotion of Bone Regeneration?

**DOI:** 10.3390/molecules26206131

**Published:** 2021-10-11

**Authors:** Wei Cao, Jianfeng Jin, Gang Wu, Nathalie Bravenboer, Marco N. Helder, Janak L. Pathak, Behrouz Zandieh-Doulabi, Jolanda M. A. Hogervorst, Shingo Matsukawa, Lester C. Geonzon, Rommel G. Bacabac, Engelbert A. J. M. Schulten, Jenneke Klein-Nulend

**Affiliations:** 1 Department of Oral Cell Biology, Academic Centre for Dentistry Amsterdam (ACTA), University of Amsterdam and Vrije Universiteit Amsterdam, Amsterdam Movement Sciences, 1081 LA Amsterdam, The Netherlands; w.cao@acta.nl (W.C.); j.jin@acta.nl (J.J.); g.wu@acta.nl (G.W.); bzandiehdoulabi@acta.nl (B.Z.-D.); jma.hogervorst@acta.nl (J.M.A.H.); 2 Department of Oral and Maxillofacial Surgery/Oral Pathology, Amsterdam University Medical Centers and Academic Centre for Dentistry Amsterdam (ACTA), Vrije Universiteit Amsterdam, Amsterdam Movement Sciences, 1081 HV Amsterdam, The Netherlands; m.helder@amsterdamumc.nl (M.N.H.); eajm.schulten@amsterdamumc.nl (E.A.J.M.S.); 3 Department of Clinical Chemistry, Amsterdam University Medical Centers, Vrije Universiteit Amsterdam, Amsterdam Movement Sciences, 1081 HV Amsterdam, The Netherlands; n.bravenboer@amsterdamumc.nl; 4 Guangzhou Key Laboratory of Basic and Applied Research of Oral Regenerative Medicine, Affiliated Stomatology Hospital of Guangzhou Medical University, Guangzhou 511436, China; j.pathak@gzhmu.edu.cn; 5 Department of Food Science and Technology, Tokyo University of Marine Science and Technology, Tokyo 113-8654, Japan; Matsukaw@kaiyodai.ac.jp; 6 School of Life and Environmental Science, University of Tsukuba, 1-1-1 Tennodai, Tsukuba 305-8572, Japan; lestergeonzon@gmail.com; 7 Department of Physics, Medical Biophysics Group, University of San Carlos, Cebu City 6000, Philippines; rgbacabac@gmail.com

**Keywords:** cell-based bone tissue engineering, bone defect, bone regeneration, κ-carrageenan, MC3T3-E1 pre-osteoblast, cell proliferation, osteogenic differentiation

## Abstract

Current cell-based bone tissue regeneration strategies cannot cover large bone defects. K-carrageenan is a highly hydrophilic and biocompatible seaweed-derived sulfated polysaccharide, that has been proposed as a promising candidate for tissue engineering applications. Whether κ-carrageenan can be used to enhance bone regeneration is still unclear. In this study, we aimed to investigate whether κ-carrageenan has osteogenic potential by testing its effect on pre-osteoblast proliferation and osteogenic differentiation in vitro. Treatment with κ-carrageenan (0.5 and 2 mg/mL) increased both MC3T3-E1 pre-osteoblast adhesion and spreading at 1 h. K-carrageenan (0.125–2 mg/mL) dose-dependently increased pre-osteoblast proliferation and metabolic activity, with a maximum effect at 2 mg/mL at day three. K-carrageenan (0.5 and 2 mg/mL) increased osteogenic differentiation, as shown by enhanced alkaline phosphatase activity (1.8-fold increase at 2 mg/mL) at day four, and matrix mineralization (6.2-fold increase at 2 mg/mL) at day 21. K-carrageenan enhanced osteogenic gene expression (*Opn*, *Dmp1*, and *Mepe*) at day 14 and 21. In conclusion, κ-carrageenan promoted MC3T3-E1 pre-osteoblast adhesion and spreading, metabolic activity, proliferation, and osteogenic differentiation, suggesting that κ-carrageenan is a potential osteogenic inductive factor for clinical application to enhance bone regeneration.

## 1. Introduction

The current clinical challenges of cell-based bone tissue regeneration are related to the limited healing capacity of bone tissue, since current bone tissue regeneration cannot cover all types of bone defect, especially when the bone defect is large and complex [1]. Common bone defects can have a systemic or local cause such as congenital abnormalities, general diseases, inflammation, or traumatic injuries such as accidents or surgical treatments [2]. Although autologous bone graft is still the “gold standard”, it has some disadvantages, such as donor site morbidity and limited volume of bone graft [3]. To resolve these clinical challenges, cell-based bone tissue engineering techniques utilize both osteogenic cells and biomaterials [4]. Currently available synthetic bone grafts are biocompatible and osteoconductive, but the majority of these biomaterials lack osteoinductivity. Ideal synthetic bone grafts should be designed to promote the adhesion, proliferation, and osteogenic differentiation of loaded precursor cells or migrated endogenous stem cells [5]. Biomaterials coated with various growth factors, proteins, and/or drugs promote the expansion and osteogenic differentiation of precursor cells [3]. However, these growth factors or drugs might cause local and systemic adverse effects that hinder the clinical application for bone regeneration [5]. Therefore, the search for safe and effective biomaterials to promote bone regeneration is still ongoing.

Carrageenan is a natural polysaccharide extracted from diverse red seaweeds with abundant sulfate groups [6]. The location of the sulfate groups and the proportion of 3,6-anhydrogalactose differ between carrageenan types such as kappa (κ)-carrageenan, iota (ι)-carrageenan, and lambda (λ)-carrageenan [7]. K-carrageenan and ι-carrageenan are commonly used for tissue engineering-related applications. K-carrageenan forms thermally reversible brittle and stiff gels, while ι-carrageenan forms elastic and soft gels [8]. λ-carrageenan does not form hydrogels and is even more fluidic compared to the other carrageenan isoforms. Engineered bone tissue requires certain mechanical and structural properties for proper functioning. Based on its properties, κ-carrageenan is the most appropriate carrageenan for bone tissue engineering applications. K-carrageenan also interacts synergistically with some polymannans, e.g., locust bean gum and konjac, to form strong cohesive gels [9]. Carrageenan is a common food additive providing some functional characteristics that can be used to gel, thicken, and stabilize food products [10]. Gelation properties, mechanical strength, and structural similarity with glycosaminoglycan (GAG) components chondroitin-4-sulphate and dermatan sulphate ensure the application of κ-carrageenan in tissue engineering [11]. The extracellular matrix components, GAGs, are naturally found in human bone and cartilage. The structural similarity between carrageenan and GAGs could be responsible for improved cell adhesion and proliferation [12]. Carrageenan has both chondrogenic and osteogenic potential [13]. Gelatin κ-carrageenan sericin hydrogel composites improve cell viability of cryopreserved SaOS-2 cells [14]. Owing to its attractive physicochemical properties, κ-carrageenan has been developed into a versatile biomaterial vehicle for drug delivery [15]. It has been used as a biomaterial, and its biocompatibility has been widely proven [7]. Aside from common applications such as food additives, carrageenans also exhibit beneficial biological properties, e.g., anticoagulant, antiviral, antioxidative, anticancer, anti-inflammation, and antihyperlipidemic properties. To develop the ideal interface for biomimetic mineralization, an effective strategy is to explore organic/inorganic composite that can mimic the nature of bone. Carrageenan is a naturally linear polysaccharide of about 25,000 galactose derivatives, consisting of highly sulfated alternating 3-linked-β-d-galactopyranose and 4-linked-R-d-galactopyranose units [16]. It exhibits very good biocompatibility and has been widely used in the food, pharmaceutical, and cosmetic industries. Since it has abundant sulfate groups, carrageenan has the potential to mimic the charged proteins present in the extracellular matrix [17]. However, the effect of κ-carrageenan on osteoblast precursor cell adhesion, proliferation, and osteogenic differentiation still needs to be investigated.

This study aimed to investigate the effect of κ-carrageenan on adhesion, metabolic activity, proliferation, and osteogenic differentiation of MC3T3-E1 pre-osteoblasts. The results of this study elucidate the possible application of κ-carrageenan in the development of bone regenerative biomaterials.

## 2. Results

### 2.1. Effect of K-Carrageenan on Cell Adhesion

**Figure 1:**
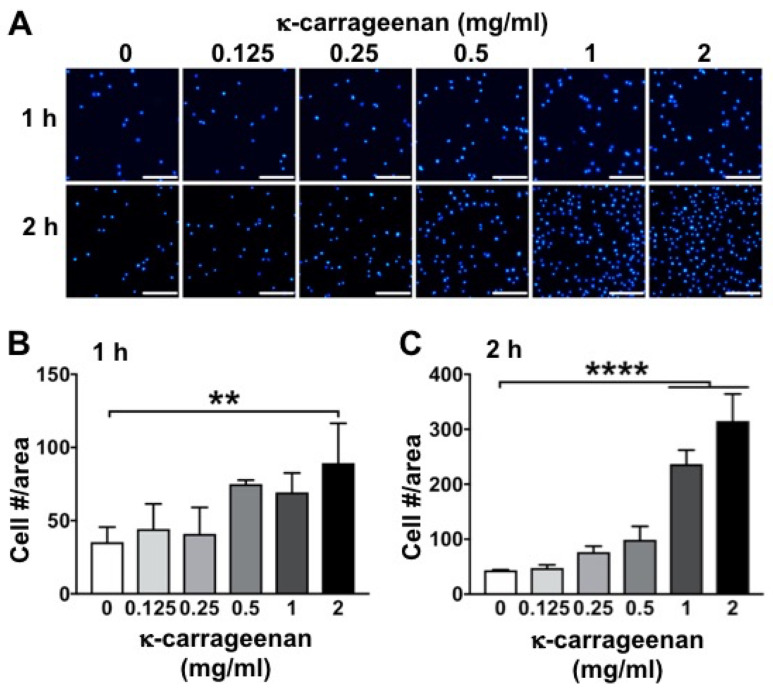
K-carrageenan-enhanced MC3T3-E1 pre-osteoblast adhesion at 1 and 2 h. (**A**) DAPI-stained nuclei of adhered cells. (**B**) Quantification of the number of adhered cells at 1 h. (**C**) Quantification of the number of adhered cells at 2 h. Values are mean ± SD from 3 independent experiments. Significant effect of κ-carrageenan, ** *p* < 0.01, **** *p* < 0.0001. Bar: 200 μm.

The number of MC3T3-E1 pre-osteoblasts adhered per well increased with increasing concentrations of κ-carrageenan after 1 and 2 h (Figure 1A). At 1 h, κ-carrageenan at 2 mg/mL significantly stimulated cell adhesion by 2.53-fold compared to untreated cells (Figure 1B). At 2 h, κ-carrageenan at 1 and 2 mg/mL enhanced cell adhesion by 5.42 and 6.61-fold, respectively (Figure 1C).

### 2.2. Effect of K-Carrageenan on Cell Area and Morphology

**Figure 2:**
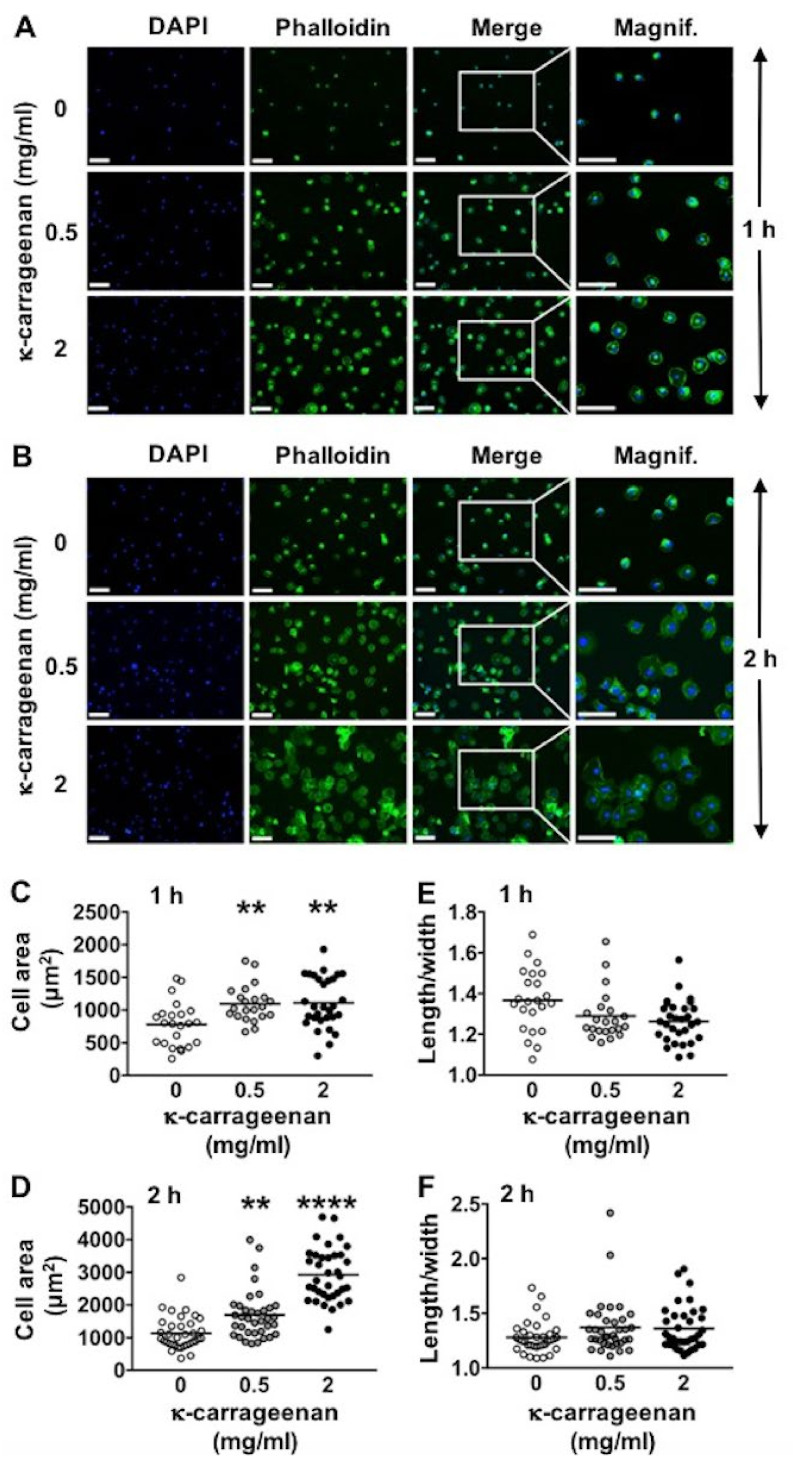
K-carrageenan enhanced the spreading of adhered MC3T3-E1 pre-osteoblasts at 1 and 2 h. (**A**) DAPI-stained nuclei and phalloidin-stained F-actin in MC3T3-E1 pre-osteoblasts at 1 h, and (**B**) at 2 h. (**C**) Cell surface area at 1 h, and (**D**) at 2 h. (**E**) Cell length/width ratio at 1 h, and (**F**) at 2 h. Values are mean ± SD from 3 independent experiments. Significant effect of κ-carrageenan, ** *p* < 0.05, **** *p* < 0.0001. Bar: 200 μm. Magnification: 2×.

K-carrageenan enhanced MC3T3-E1 pre-osteoblast adhesion and spreading in a dose-dependent manner at 1 and 2 h (Figure 2A,B). At 1 h, κ-carrageenan at 0.5 and 2 mg/mL significantly enhanced the cell surface area by 1.52 and 1.55-fold, respectively (Figure 2C). At 2 h, κ-carrageenan at 0.5 and 2 mg/mL significantly enhanced the cell surface area by 1.45-fold and 1.91-fold, respectively (Figure 2D). K-carrageenan treatment did not affect the cell length/width ratio (Figure 2E,F).

### 2.3. Effect of K-Carrageenan on Paxillin Protein Expression

**Figure 3:**
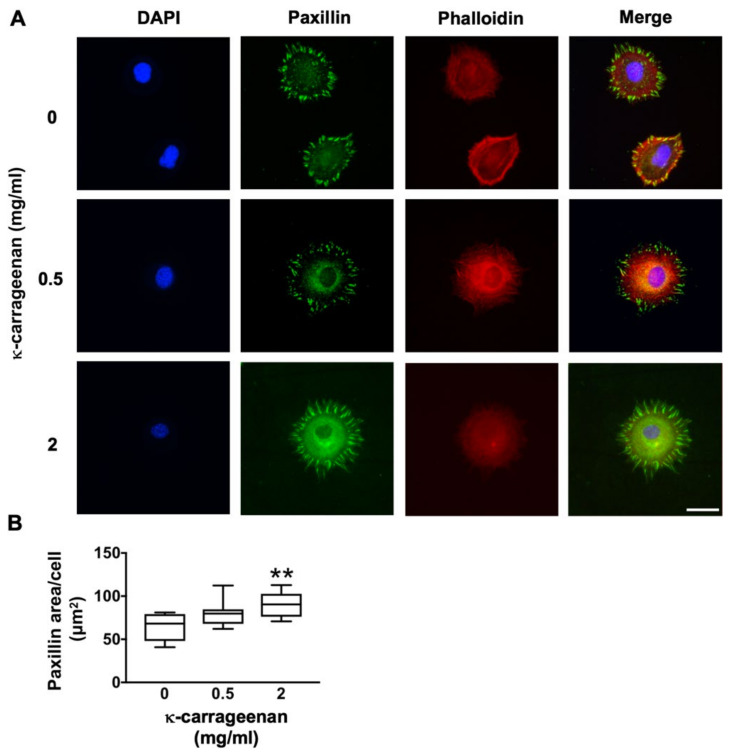
Effect of κ-carrageenan on paxillin expression and distribution in MC3T3-E1 pre-osteoblasts at 2 h. (**A**) Cells stained for DAPI/phalloidin (blue/red) and paxillin (green). Bar: 50 µm. (**B**) Quantification of paxillin area in untreated control and κ-carrageenan-treated cells. Significant effect of κ-carrageenan, ** *p* < 0.01.

Immunofluorescence staining of paxillin revealed clear clusters at the cell boundary of untreated control MC3T3-E1 pre-osteoblasts (Figure 3A). Treatment with κ-carrageenan resulted in more paxillin dots, resembling short rods (Figure 3B). This was confirmed by a significant 1.39-fold increase in fluorescent paxillin area per cell after 2 h treatment with κ-carrageenan at 2 mg/mL.

### 2.4. Effect of K-Carrageenan on Cell Migration

**Figure 4:**
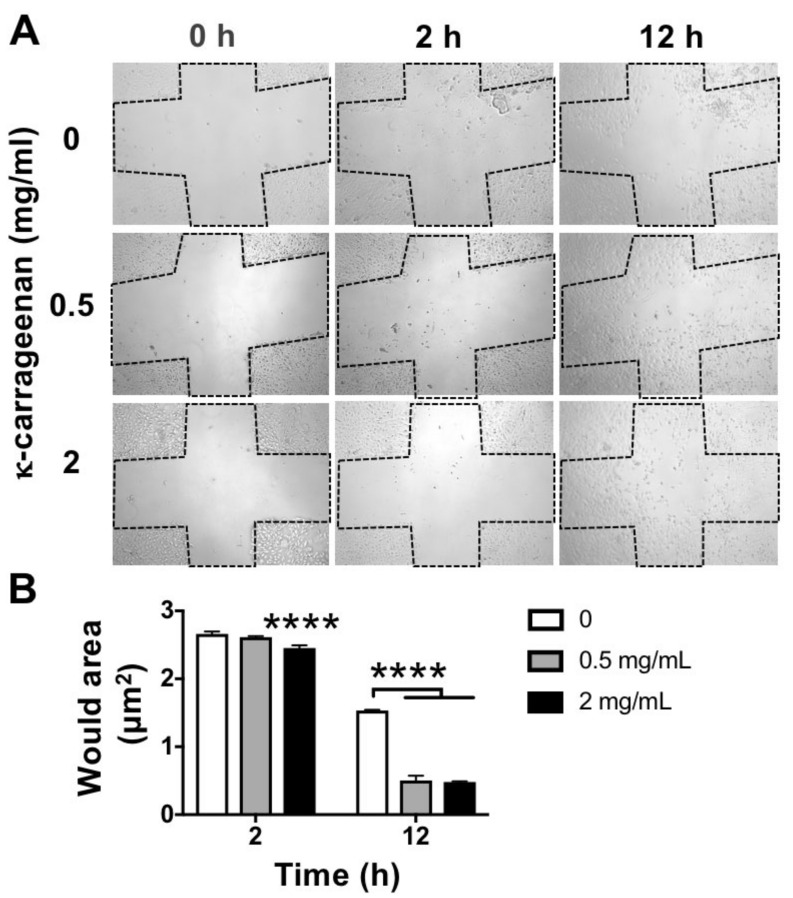
Effect of κ-carrageenan on migration in MC3T3-E1 pre-osteoblasts at 2 and 12 h. (**A**) Images of wound area with and without κ-carrageenan. (**B**) Quantification of wound area. Values are mean ± SD from three independent experiments. Significant effect of κ-carrageenan, **** *p* < 0.0001.

At 12 h, the wound area was significantly reduced after treatment with 2 mg/mL κ-carrageenan (Figure 4A). K-carrageenan at 2 mg/mL significantly stimulated cell migration by 3.19-fold compared to untreated controls. At 2 and 12 h, κ-carrageenan at 0.5 and 2 mg/mL did promote cell migration compared to untreated controls (Figure 4B).

### 2.5. Effect of K-Carrageenan on Cell Metabolic Activity and Proliferation

**Figure 5:**
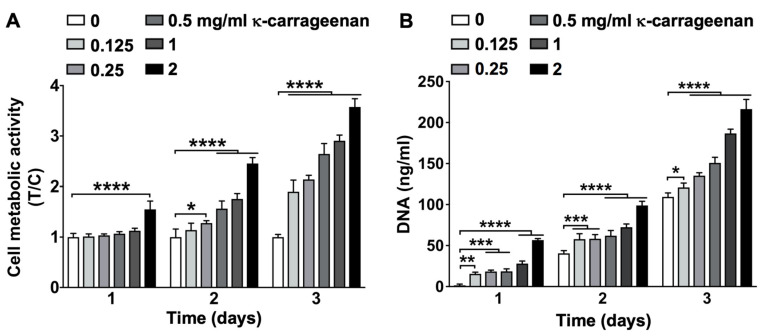
K-carrageenan enhanced MC3T3-E1 pre-osteoblast metabolic activity and proliferation from day 1 to day 3. (**A**) Presto-blue cell metabolic activity. Data are expressed as κ-carrageenan-treated-over-control (T/C) ratio. (**B**) Total cellular DNA content. Values are mean ± SD from 4 independent experiments. Significant effect of κ-carrageenan, * *p* < 0.05, ** *p* < 0.01, *** *p* < 0.001, **** *p* < 0.0001.

The effect of κ-carrageenan on MC3T3-E1 pre-osteoblast metabolic activity and proliferation was dose-dependent. MC3T3-E1 pre-osteoblast metabolic activity was most significantly enhanced after treatment with 2 mg/mL κ-carrageenan from day 1 to 3 (maximum 3.6-fold increase, day 3) (Figure 5A). Cell proliferation was assessed by measuring cellular DNA content, which was most significantly enhanced by treatment with κ-carrageenan at 2 mg/mL from day 1 to 3 (maximum increase 50.92-fold, day 1) (Figure 5B).

### 2.6. Effect of K-Carrageenan on Osteogenic Gene Expression

**Figure 6:**
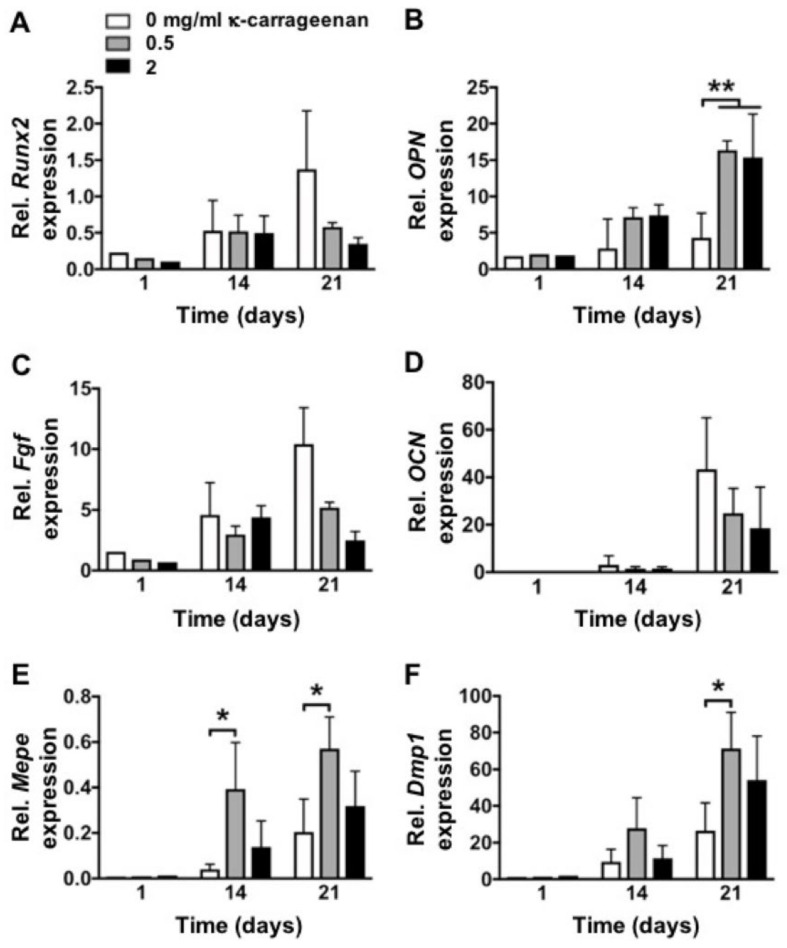
K-Carrageenan enhanced expression of osteogenic markers in MC3T3-E1 pre-osteoblasts at day 1, 14, and 21. Expression pattern of (**A**) *Runx2*, (**B**) *Opn*, (**C**) *Fgf2*, (**D**) *Ocn*, (**E**) *Mepe*, and (**F**) *Dmp1*. Values are mean ± SD from three independent experiments. Significant effect of κ-carrageenan, * *p* < 0.05, ** *p* < 0.01.

K-carrageenan treatment significantly upregulated mRNA expression of osteogenic differentiation markers (*Opn*, *Dmp1*) compared to untreated controls (Figure 6). In addition, *Mepe* expression was significantly upregulated by treatment with κ-carrageenan at day 14 and 21 compared to untreated controls (* *p* < 0.05).

### 2.7. Effect of K-Carrageenan on ALP Activity

**Figure 7:**
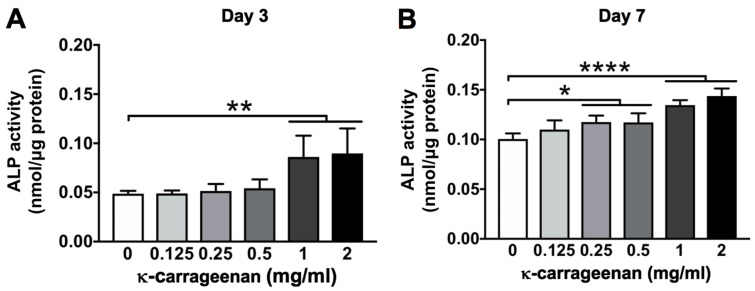
K-carrageenan enhanced ALP activity of MC3T3-E1 pre-osteoblasts at day 3 and 7. ALP activity at (**A**) day 3, and (**B**) day 7. Values are mean ± SD from 4 independent experiments. Significant effect of κ-carrageenan, * *p* < 0.05, ** *p* < 0.01, **** *p* < 0.0001. ALP, alkaline phosphatase.

ALP activity in MC3T3-E1 pre-osteoblasts was measured at day 4 and 7 (Figure 7A,B). K-carrageenan at 1 and 2 mg/mL increased ALP activity at day 4. K-carrageenan at 0.25, 0.5, 1, and 2 mg/mL enhanced ALP activity at day 7 compared with untreated controls, with 2 mg/mL showing the strongest effect.

### 2.8. Effect of K-Carrageenan on Mineralized Extracellular Matrix Production

**Figure 8:**
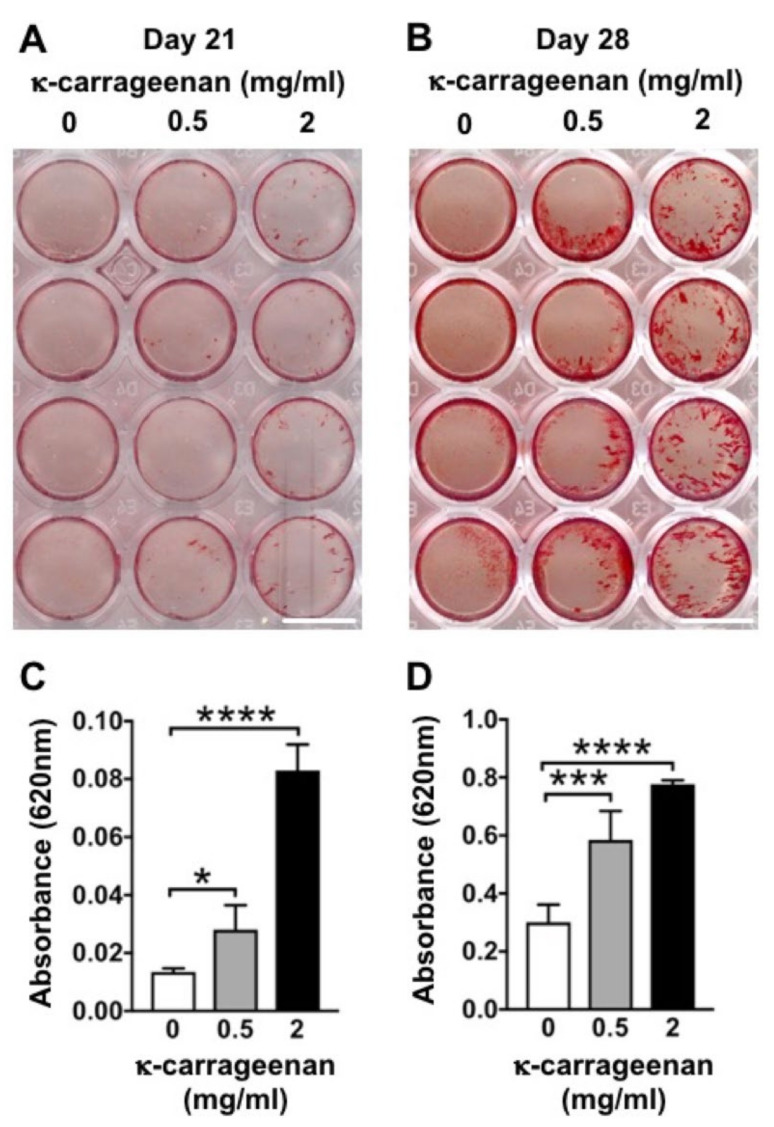
K-carrageenan promoted matrix mineralization in MC3T3-E1 pre-osteoblasts at day 21 and 28. (**A**,**B**) Alizarin red-stained mineralized matrix. (**C**,**D**). Quantification of alizarin red-stained mineralized matrix. Values are mean ± SD from four independent experiments. Significant effect of κ-carrageenan, * *p* < 0.05, *** *p* < 0.001, **** *p* < 0.0001. Bar: 1 cm.

K-carrageenan at 0.5 and 2 mg/mL increased matrix mineralization in MC3T3-E1 pre-osteoblasts at day 21 and 28 (Figure 8). K-carrageenan treatment resulted in more mineralized matrix at day 21 (1.8-fold increase, at 0.5 mg/mL; 4.5-fold increase, at 2 mg/mL) and at day 28 (1.6-fold increase, at 0.5 mg/mL; 2.2-fold increase, at 2 mg/mL) compared to untreated controls.

## 3. Discussion

Biomaterials used for bone tissue engineering should be highly biocompatible, mechanically rigid, and biodegradable as well. Surface topography and surface charge also determine the efficacy of biomaterials for bone regeneration. These basic properties can facilitate cellular activities, such as adhesion, migration, proliferation, and osteogenic differentiation [5]. Recently, κ-carrageenan, a natural polymer extracted from red seaweed, has been shown to exhibit tissue regenerative potential [6]. The three isoforms of carrageenan, i.e., λ-, κ-, and ι-carrageenan, possess varying water solubilities. λ-carrageenan is the most soluble and does not form hydrogels, while ι- and κ-carrageenan will. The ι-isoform, however, creates much softer hydrogels compared to the κ-variant. So in fact, the κ-carrageenan is sufficiently strong to be used in bone tissue engineering. We found that κ-carrageenan significantly increased migration, early cell adhesion, spreading, and proliferation of MC3T3-E1 pre-osteoblasts. In addition, κ-carrageenan promoted ALP activity, matrix mineralization, and osteogenic gene expression, e.g., *Runx-2*, *Ocn*, *Opn*, *Fgf2*, *Dmp1*, and *Mepe*. These results suggest that κ-carrageenan could be applicable in bone tissue engineering.

Natural polymers such as silk fiber, chitosan, hyaluronic acid, fibrin, and alginate, have tissue regenerative potential in vitro and in vivo [16,17]. However, their clinical application is still a challenge due to associated adverse effects and limited resources. Adverse effects such as chronic inflammation and foreign body reactions can result from by-products of polymers used for clinical application. K-carrageenan is a natural polymer extracted from red seaweed, which is abundantly present in the south Asian seas [18]. Easy extraction and high natural abundance guarantee its availability for cost-effective clinical application. Both the osteogenic and the soft tissue regenerative potential of κ-carrageenan have been reported [7]. An essential feature of live cells is motility. Cell migration is involved in the conception of life, embryonic development, immune response, and many pathological processes such as cancer metastasis and inflammation [19,20]. Precursor cell chemotaxis in a defect site is essential for endogenous bone regeneration [21]. Therefore, the design of biomaterials with chemotactic potential to induce endogenous precursor cell homing in the defect site is at the center of attention in the field of bone tissue engineering [21]. In this study, κ-carrageenan at 0.5 and 2 mg/mL robustly promoted the migration of pre-osteoblasts at 24 h after seeding. Our results indicate the possible application of κ-carrageenan in biomaterials designed for endogenous bone regeneration.

Surface adhesion, spreading, proliferation, and differentiation of osteogenic cells are critical steps for successful cell-based bone tissue engineering techniques [5]. Consequently, much effort has been put into the development of a wide variety of techniques, such as calcium phosphate deposition [22] and RGD peptide adhesion on titanium surface to modify or functionalize surface properties in order to improve cell–substrate interactions [23]. In this study, κ-carrageenan promoted pre-osteoblast adhesion and spreading. K-carrageenan-functionalized graphene oxide composite enhances mineralization by hydroxyapatite deposition [24]. This composite enhances the adhesion of MC3T3-E1 pre-osteoblasts [25]. Various bone grafts and implants with similar mechanical and physicochemical properties as bone fail osseointegration due to a lack of cell adhesion properties [4]. Our data, as well as data from others [26,27], have shown that κ-carrageenan could be used as a coating to enhance precursor cell adhesion on bone grafts and implants.

K-carrageenan (<2 mg/mL), did not show any cytotoxic effect on pre-osteoblasts, indicating its biocompatibility. Biomaterials should be biocompatible and non-cytotoxic for proper adhesion and spreading of migrated precursor cells [5]. K-carrageenan (<2 mg/mL) induced pre-osteoblast metabolic activity in a dose-dependent manner. Since precursor cell metabolic activity plays a key role in osteogenic differentiation [6], these results further support possible application of κ-carrageenan in bone tissue engineering. Lack of precursor cell growth causes failure of bone regeneration in large-sized bones [16,17]. An ideal biomaterial should not only be biocompatible, but also induce precursor cell growth. K-carrageenan (<2 mg/mL) induced MC3T3-E1 pre-osteoblast growth in a dose-dependent manner. An injectable hydrogel of κ-carrageenan-functionalized graphene oxide enhances fibroblast proliferation and spreading [11]. Carrageenan hydrogel serves as a scaffold for human skin-derived multipotent stromal cells and promotes skin wound healing [28]. We speculate that the positive impact of κ-carrageenan in our study on most cellular activities of pre-osteoblasts may be due to the abundant sulfate groups in κ-carrageenan, which mimic the charged proteins present in the extracellular matrix and ensure the practicality of κ-carrageenan for bone tissue engineering. However, studying the exact molecular mechanisms for the positive effects is beyond the scope of the current study, and is therefore not addressed in this study. Since κ-carrageenan is biocompatible, and induces osteoblast precursor cell metabolic activity, it could be beneficial for bone regenerative applications.

Once migrated precursor cells are attached and proliferate on the surface of a bone graft, osteogenic differentiation becomes crucial. The majority of bone grafts and implants lack osteogenic differentiation-inducing potential. Various growth factors, such as bone morphogenetic protein-2 (BMP2) and vascular endothelial growth factor (VEGF) have been used to enhance the osteogenic potential of bone grafts [29]. However, the high cost and local/systemic adverse effects of these growth factors limit their clinical application. In this study, κ-carrageenan promoted the osteogenic differentiation of pre-osteoblasts as indicated by enhanced ALP activity, matrix mineralization, and osteogenic gene expression. As can be deduced from Figure 6, κ-carrageenan induces maturity of osteoblasts as shown by upregulation of *Opn*, *Mepe*, and *Dmp1*. In particular, the latter are osteocytic markers showing the acquirement of an osteocyte-like phenotype. It is worth noting that Opn is an osteoblast-produced adhesive molecule which appears markedly upregulated due to the interaction with κ-carrageenan. Carrageenan nanocomposite hydrogel incorporated with whitlockite nanoparticles and dimethyloxallylglycine enhances osteoblast gene expression, e.g., *Runx2*, *COLIα1*, and *Opn* [30]. K-carrageenan-doped collagen-hydroxyapatite composite shows similar structural characteristics with natural bone [31]. K-carrageenan/silk fibroin bioactive composite scaffolds have been developed for bone regeneration applications [32]. Our data support the possible application of κ-carrageenan in the development of biomaterials for bone tissue engineering applications. Although the safety of carrageenan is widely recognized, some in vitro studies [18,33] reported that carrageenan may play a role in the toll-like receptor (TLR) pathway by binding to TLR-4, and that carrageenan causes inflammation and induces expression of proinflammatory chemokines and cytokines, e.g., IL-8, CCL2, IL6, and TNF-α in human intestinal cell lines [34]. Therefore, the possible application of carrageenan in tissue engineering, and the related regulatory pathways involved still need further investigation.

We conclude that our results indicate a possible role of κ-carrageenan in pre-osteoblast adhesion, spreading, migration, metabolic activity, proliferation, and osteogenic differentiation. This study fully explored the influence of κ-carrageenan on cell function from different aspects that are needed for bone regeneration. The current results suggest that κ-carrageenan might be a promising factor to functionalize bone graft and for enhanced osseointegration of implants. However, these findings should be further verified using in vivo bone regeneration models.

## 4. Materials and Methods

### 4.1. Preparation of K-Carrageenan

K-carrageenan (See Supplementary Materials) was kindly supplied by Tokyo Chemical Industry Co., Ltd. (Tokyo, Japan), and was prepared as described earlier [7]. Samples were dialyzed first against NaCl solution, and second against deionized water, to obtain Na^+^ type κ-carrageenan, which was freeze-dried. Na^+^ and K^+^ concentrations of the dialyzed carrageenan were 0.4% and 0.11%, respectively, as determined by inductively coupled plasma atomic emission. No Mg^+^ or Ca^2+^ was detected in the sample following dialysis. The freeze-dried pure κ-carrageenan (100%, wt/vol) was dissolved in deionized water at 28 mg/mL (stock solution) under stirring overnight at room temperature, sterilized by heating for 2 min to 90 °C, and stored at 4 °C. Dialysed κ-carrageenan was frozen at ~60 °C overnight and freeze-dried at ~70 °C using a vacuum freeze-dryer (Neocool, Yamato Scientific, Tokyo, Japan). K-carrageenan was added to MC3T3-E1 pre-osteoblast cultures (see below) at 0.125, 0.25, 0.5, 1, and 2 mg/mL α-Minimal Essential Medium (α-MEM; Gibco, Paisly, UK) supplemented with 10% fetal bovine serum (FBS; Gibco), 100 U/mL penicillin (Sigma-Aldrich, St. Louis, MO, USA), 100 μg/mL streptomycin (Sigma-Aldrich), and 1.25 μg/mL fungizone (Gibco).

### 4.2. MC3T3-E1 Pre-Osteoblast Culture and Osteogenic Differentiation

MC3T3-E1 pre-osteoblasts were obtained from the American Type Culture Collection (ATCC, Manassas, VA, USA). For experiments, cells of passage 12–25 were used. Cells were grown and maintained in 75 cm^2^ culture flasks (Greiner, Bio-one, Alphen a/d Rijn, The Netherlands) containing α-MEM supplemented with 10% FBS and antibiotics in a humidified atmosphere with 5% CO_2_ in air at 37 °C. The medium was changed every 3 days. After reaching ~80% confluency, cells were detached using 0.25% trypsin (Gibco, Invitrogen, Waltham, MA, USA) and 0.1% ethylenediaminetetraacetic acid (EDTA; Merck, Darmstadt, Germany) in phosphate-buffered saline (PBS; Gibco) at 37 °C. Cells were then resuspended in α-MEM with 10% FBS and antibiotics, seeded in 96, 48, or 24-well culture plates (Greiner), and cultured for different periods, from 1 h up to 28 days, dependent on the outcome parameter measured (see below). For determination of alkaline phosphatase (ALP) activity and matrix mineralization, 0.1 mg/mL ascorbic acid (Sigma-Aldrich) and 10 mM β-glycerophosphate (phosphate donor; Sigma-Aldrich) were added to the culture medium.

### 4.3. Cell Adhesion

MC3T3-E1 pre-osteoblasts were seeded at 2 × 10^4^ cells/well in 24-well plates (Greiner) and incubated for 1 or 2 h without or with κ-carrageenan (0.125, 0.25, 0.5, 1, or 2 mg/mL). After incubation, the medium was removed and the cells were washed twice with PBS and fixed in 4% paraformaldehyde for 15 min at room temperature. After washing twice with deionized water, 200 μL 4,6-diamidino-2-phenylindole (DAPI; Sigma, Los Angeles, CA, USA) solution (0.1 µg/mL) was added to the wells for 10 min to stain the cell nuclei (blue). Fluorescence microscopy (Leica, Wetzlar, Germany) was used to visualize the cells at 496/516 nm wavelength, and Image J software (National Institutes of Health, Bethesda, MD, USA) was used for cell counting. In each well, three areas of interest, measuring 1920 × 1440 pixels (872 × 654 μm) were defined, i.e., one area in the center of the well, and two areas evenly spaced from the center of the well in opposite directions. The number of cells in every area of interest was counted using a cell counter plugin for Image J. The cell number per area was calculated from the number of cell nuclei acquired in the selected area.

### 4.4. Cell Area and Morphology

After cell culture and fixation in 4% paraformaldehyde, as described above under “cell adhesion”, the fixated cells were washed twice with deionized water, and 200 µL FITC-phalloidin (100 nM; Sigma, Los Angeles, CA, USA) solution was added during 1 h for cytoskeletal staining. After washing twice with deionized water, 200 μL DAPI solution (0.1 µg/mL) was added for 10 min to the wells to stain the nuclei (blue). Fluorescence microscopy (Leica, Wetzlar, Germany) was used to visualize the cell nuclei at 496/516 nm wavelength, and Image J software was used for cell counting. Fluorescence microscopy was also employed to visualize the actin cytoskeleton at 500/550 nm wavelength, and Image J software was used for cell area determination [4]. In each well, three areas of interest, measuring 1920 × 1440 pixels (872 × 654 μm) were defined, i.e., one area in the center of the well, and two areas evenly spaced from the center of the well in opposite directions. The number of cells in every area of interest was counted using a cell counter plugin for Image J. The cell number per area was calculated from the cell nuclei number acquired in the selected area. The cell surface area of all cells (total cell surface area) in the selected area was calculated using a cell surface plugin for Image J. The cell surface area per cell was calculated using an Analyze Particles cell plugin in Image J. To investigate cell morphology, the center of each area chosen above was magnified, and Image J software was used to analyze cell area, length, and width.

### 4.5. Paxillin Immunofluorescence Staining

After treatment without or with κ-carrageenan (0.5 and 2 mg/mL) for 2 h, MC3T3-E1 pre-osteoblasts were fixed with 4% paraformaldehyde solution at 37 °C for 15 min, treated with 0.2% TritonX-100 (Sigma) for 15 min, and non-essentially bound substances blocked in 5% bovine serum albumin (BSA) for 30 min. Expression of phospho-paxillin (p-paxillin) was analyzed by immunofluorescence staining using rhodamine-phalloidin cytoskeleton dye (Invitrogen, Fisher Scientific, Carlsbad, CA, USA) and p-paxillin pTy31 polyclonal rabbit IgG (ab32084, Abcam, Cambridgeshire, UK). The secondary antibody used was Alexa Fluor-488 goat anti-rat IgG (Abcam). Nuclei were stained with 1 µg/mL DAPI (Sigma). After glycerol mounting, cell imaging was performed by laser scanning confocal microscopy (LSCM; Nikon, A1R/A1, Tokyo, Japan). Fluorescence microscopy was also used to visualize paxillin at 488 nm wavelength, and Image J software was used for paxillin quantification.

### 4.6. Wound Healing Scratch Assay for Cell Migration

MC3T3-E1 pre-osteoblasts were cultured at 5 × 10^4^ cells/well in 24-well plates (Greiner) 12 h prior to scratching. A 200 μL pipette tip was firmly pressed against the top of the tissue culture plate to swiftly create a vertical wound through the cell monolayer. After treatment without or with κ-carrageenan (0.5 and 2 mg/mL) for 2 and 12 h, the wound area was captured using microscopy (Leica, Wetzlar, Germany) and Image J software to quantify the wound area.

### 4.7. Cell Metabolic Activity

To assess cell metabolic activity, MC3T3-E1 pre-osteoblasts were seeded at 1 × 10^5^ cells/well in 48-well plates (Greiner) and cultured up to 3 days without or with κ-carrageenan (0, 0.125, 0.25, 0.5, 1, or 2 mg/mL). The medium was removed, cells were washed with PBS, and α-MEM with 10% FBS and antibiotics was added. The PrestoBlue^TM^ Assay (Invitrogen Corporation, Carlsbad, CA, USA) was used to evaluate cell metabolic activity according to the manufacturer’s instructions. In short, PrestoBlue^TM^ reagent was added to the cell culture (10%, vol/vol), followed by 30 min incubation in a 5% CO_2_ in an air incubator with a humidified atmosphere at 37 °C. The medium was harvested (100 μL/well) and transferred into a 96-well black microplate (Greiner, Bio-one, Alphen a/d Rijn, The Netherlands). Fluorescence intensity was determined at a wavelength of 560 nm (excitation) and 590 nm (emission), and quantified using a Multiskan^TM^ FC Microplate Photometer (Thermo Fisher Scientific, Waltham, MA, USA). Prestoblue^TM^ fluorescence was linearly associated to DNA content (data not shown).

### 4.8. DNA Content

MC3T3-E1 pre-osteoblast proliferation was assessed by DNA content quantification. Cells were seeded at 1 × 10^3^ cells/well in 96-wells plates (Greiner), and cultured up to three days without or with κ-carrageenan (0, 0.125, 0.25, 0.5, 1, or 2 mg/mL). The cell lysate was collected using a lysis buffer, and DNA content per well was determined with the Cyquant Cell Proliferation Assay (Molecular Probes, Eugene, OR, USA) according to the manufacturer’s protocol. Absorption was read at 480 nm (excitation) and 520 nm (emission) in a microplate reader (Synergy, BioTek^TM^, Winooski, VT, USA).

### 4.9. Osteogenic Gene Expression

Total RNA was isolated from the MC3T3-E1 pre-osteoblasts using an Invitrogen RNA isolation kit (Invitrogen, Carlsbad, CA, USA). cDNA synthesis was performed using 0.5–1 μg total RNA in 20 μL reaction mix consisting of 5 units Transcriptor Reverse Transcriptases (Roche Diagnostics, Basel, Switzerland), 0.08 A_260_ units of random primers (Roche Diagnostics), 1 mM of each dNTP (Invitrogen), and 1× concentrated Transcriptor RT reaction buffer (Roche Diagnostics). Real-time PCR (RT-PCR) reactions were performed using the LightCycler^®^ 480 SYBR green I Master reaction mix according to the manufacturer’s instructions (Roche Diagnostics) in a Light Cycler 480 (Roche Diagnostics), and relative housekeeping gene expression (PBGD) and relative target gene expression, such as runt-related transcription factor 2 (*Runx2*), osteocalcin (*Ocn*), fibroblast growth factor 2 (*Fgf2*), dentin matrix protein 1 (*Dmp1*), and osteopontin (*Opn*) were determined. The primers (Invitrogen) used for RT-PCR are listed in Table 1. The values of target gene expression were normalized for *PBGD* gene expression.

### 4.10. Alkaline Phosphatase (ALP) Activity

To assess the osteogenic phenotype of MC3T3-E1 pre-osteoblasts after 4 and 7 days of culture without or with κ-carrageenan, ALP activity was measured. Cells were seeded at 1 × 10^5^ cells/well in 48-well plates, and cultured for 4 or 7 days to determine ALP activity and protein content in the cell lysate. P-nitrophenyl phosphate (Merck, Darmstadt, Germany) at pH 10.3 was used as a substrate to determine ALP activity. The absorbance was read at 405 nm in a microplate reader (Synergy). The amount of protein was determined using a BCA Protein Assay Reagent kit (Thermo Fisher Scientific, Rockford, IL, USA), and the absorbance was read at 540 nm with a microplate reader (Synergy). The ALP activity was expressed as nmol/μg protein.

### 4.11. Alizarin Red Staining and Mineralized Nodule Quantification

Matrix mineralization was analyzed by alizarin red staining of MC3T3-E1 pre-osteoblasts. Cells were seeded at 1 × 10^5^ cells/well in 48-well plates, and incubated without or with κ-carrageenan (0.5 or 2 mg/mL). Cells were fixed with 4% paraformaldehyde for 15 min, followed by rinsing with deionized water. Two-hundred μL of 2% alizarin red solution in water, pH 4.3 (Alizarin Red S, Sigma-Aldrich, Los Angeles, CA, USA), was added per well for 30 min at room temperature. Then cells were washed with deionized water, and mineralization was quantified by dissolving the (red) mineralized matrix in 10% (vol/vol) cetylpyridinium chloride (Sigma, Los Angeles, CA, USA) in 10 mM sodium phosphate solution (Sigma). Wells were de-stained for 1 h in 200 μL cetylpyridinium chloride solution on a rocking table, and the absorbance was read at 620 nm with a Multiskan FC (Thermo Fisher Scientific).

### 4.12. Statistical Analysis

Data are presented as mean ± standard deviation (SD). Data were analyzed using Graphpad Prism^®^ 7.0 (GraphPad Software Inc., La Jolla, CA, USA). One-way analysis of variance (ANOVA) with Bonferroni’s post hoc test was used to test differences between groups. A *p*-value < 0.05 was considered statistically significant.

## Figures and Tables

**Table 1:** Primer sequences used for real-time PCR.

Target Gene	Primer Sequence	
*Runx2*	Forward Reverse	ATGCTTCATTCGCCTCAC ACTGCTTGCAGCCTTAAAT
*Ocn*	Forward Reverse	CAGACACCATGAGGACCATCTT GGTCTGATAGCTCGTCACAA
*Fgf2*	Forward Reverse	GGCTTCTTCCTGCGCATCCA TCCGTGACCGGTAAGTATTG
*Dmp1*	Forward Reverse	CGGCTGGTGGACTCTCTAAG CGGGGTCGTCGCTCTGCATC
*Opn* *Mepe*	Forward Reverse Forward Reverse	AGTGATGAAAGATGGGCAACT TCTGGACCATCTTCTTGCTGA GGAGCACTCACTACCTGAC TAGGCACTGCCACCATGT

*Runx2*, Runt-related transcription factor-2; *Ocn*, osteocalcin; *Fgf2*, fibroblast growth factor-2; *Dmp1*, dentin matrix protein-1; *Opn*, osteopontin; *Mepe*, matrix extracellular phosphoprotein.

## Data Availability

The data presented in this study are openly available in Figshare at 10.6084/m9. figshare. 15156741.

## Supplementary Materials

The following are available online, Figure S1: The chemical structure of κ-carrageenan.

## Sample Availability

Samples of the compounds are not available from the authors.
