# Supplementary material for: K-Carrageenan Stimulates Pre-Osteoblast Proliferation and Osteogenic Differentiation: A Potential Factor for the Promotion of Bone Regeneration?
Source: Molecules. 2021 Oct 11;26(20):6131. doi: 10.3390/molecules26206131 (PMC8541607; doi:10.3390/molecules26206131)
Supplement: Supplementary file 1 [file molecules-26-06131-s001.zip › molecules-1362586-supplementary.pdf]

SUPPLEMENTARY FIGURE

MS #: Molecules-1362586

MS Title: Kappa-carrageenan Stimulates Pre-osteoblast Proliferation and Osteogenic Differentiation: A Potential Factor for the Promotion of Bone Regeneration?

Authors: Wei Cao, Jianfeng Jin, Gang Wu, Nathalie Bravenboer, Marco N. Helder, Janak L. Pathak, Behrouz Zandieh-Doulabi, Jolanda M.A. Hogervorst, Shingo Matsukawa, Lester C. Geonzon, Rommel G. Bacabac, Engelbert A.J.M. Schulten, Jenneke Klein-Nulend

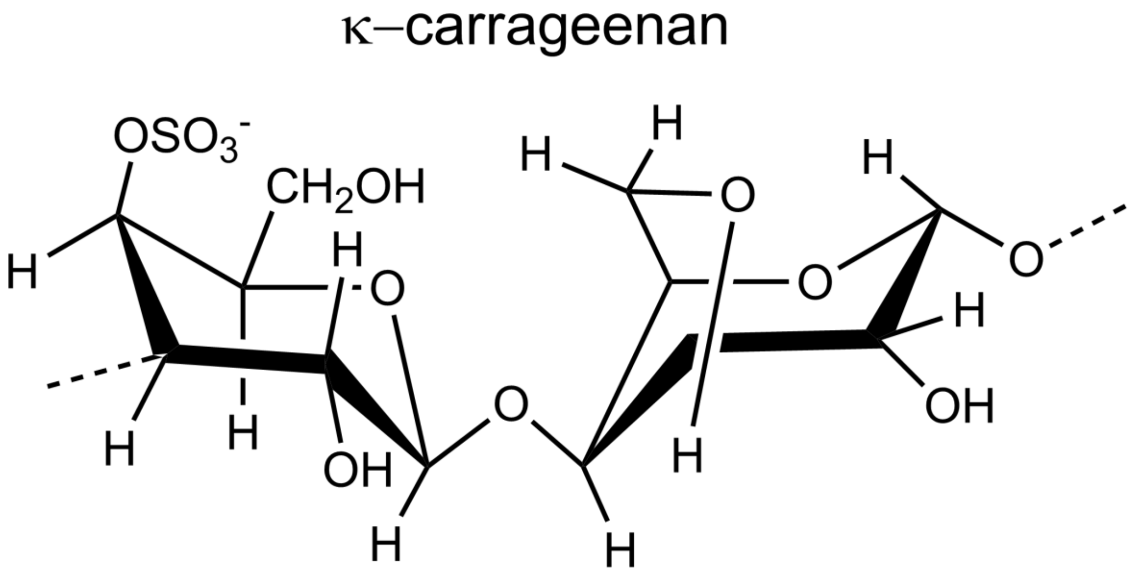

Figure S1. The chemical structure of k-carrageenan.
